# Analytical ultracentrifugation for analysis of doxorubicin loaded liposomes

**DOI:** 10.1016/j.ijpharm.2017.03.046

**Published:** 2017-05-15

**Authors:** Dora Mehn, Patrizia Iavicoli, Noelia Cabaleiro, Sven Even Borgos, Fanny Caputo, Otmar Geiss, Luigi Calzolai, François Rossi, Douglas Gilliland

**Affiliations:** a Consumer Products Safety, Directorate General Joint Research Centre, European Commission, Directorate F – Health, Consumers and Reference Materials, Via E. Fermi, I-21027 Ispra (VA), Italy; b SINTEF Materials and Chemistry, Department of Biotechnology and Nanomedicine, Sem Sælands v. 2A, N-7034 Trondheim, Norway; c University Grenoble Alpes, F38000 Grenoble, France; d CEA, LETI, Minatec Campus, F38054 Grenoble, France

**Keywords:** Doxorubicin hydrochloride (PubChem CID: 443939), Analytical ultracentrifugation, AUC, Liposome, Nanomedicine, Free drug, Size distribution

## Abstract

Analytical ultracentrifugation (AUC) is a powerful tool for the study of particle size distributions and interactions with high accuracy and resolution. In this work, we show how the analysis of sedimentation velocity data from the AUC can be used to characterize nanocarrier drug delivery systems used in nanomedicine. Nanocarrier size distribution and the ratio of free versus nanoparticle-encapsulated drug in a commercially available liposomal doxorubicin formulation are determined using interference and absorbance based AUC measurements and compared with results generated with conventional techniques. Additionally, the potential of AUC in measuring particle density and the detection of nanocarrier sub-populations is discussed as well. The unique capability of AUC in providing reliable data for size and composition in a single measurement and without complex sample preparation makes this characterization technique a promising tool both in nanomedicine product development and quality control.

## Graphical abstract

**Figure:**
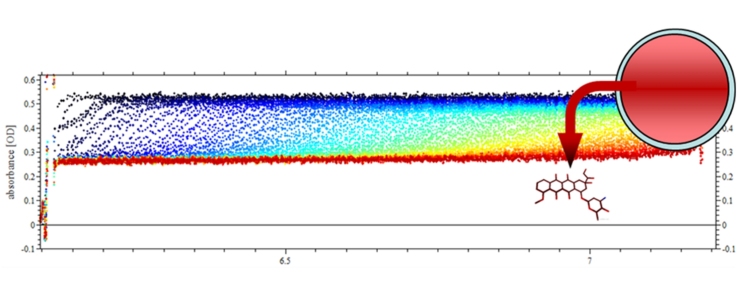

## 1 Introduction

Size makes the most obvious difference between nanomedicine products and conventional medicine. Indeed, carrier size distribution is identified as one of the key parameters determining the biodistribution and pharmacokinetics (PK) of nanoformulated drugs. Being crystallized, encapsulated or linked to a particle in the nano size-range often ensures a molecule better barrier penetration, improved bioavailability or more efficient accumulation in the target tissue as compared to the conventional free drug (Angi et al., 2014, Desai, 2012, Fuhrmann et al., 2014, Ross et al., 2015, Szabo and Zelko, 2015). Other features, like coatings inhibiting opsonization were also proven to be creditable for enhanced efficacy of nano-pharmaceuticals (Vonarbourg et al., 2006). In cancer treatment, direct targeting of the tumor tissue by linking antibodies or receptor ligands to the surface of the nanoparticles is identified − and sometimes disputed − as a trend in nanomedicine research (Lammers et al., 2016, van der Meel et al., 2013). However, the complications of loading active compounds in some promising nano-carriers (Raemdonck and De Smedt, 2015) or the most prominent success stories of nanomedicine, like Abraxane (Sparreboom et al., 2005) or Doxil (Barenholz, 2012) still highlight the significance of the free versus encapsulated (or bound) drug ratio in the biodistribution and pharmacokinetics of nanoformulated products. For example, in the case of Doxil, the decreased cumulative concentration-dependent cardiotoxicity of doxorubicin in pegylated liposomal formulation is attributed to the longer half-life of the drug inside the liposomes and decreased myocardial concentration of the free drug (Rahman et al., 2007, Tahover et al., 2015). In this context, the low concentration of the free cytotoxic drug in the nanomedicine product becomes a warranty for drug safety.

All the above mentioned parameters: size distribution, composition, coating, functionalization, drug loading, free versus encapsulated drug content are extremely important characteristics not only during the design and pilot experimental phase but also during the later production phases of nanomaterials. Measurement of these parameters is critical in scale-up processes, in monitoring batch to batch variations as part of an internal quality system as well as during drug safety evaluations by an external authority (Diou et al., 2015, Landesman-Milo and Peer, 2016, Ragelle et al., 2016).

Among the size measurement methods, dynamic light scattering (DLS) is the most widespread technique applied in nanomedicine research. The main drawback of batch mode DLS measurement, i.e. light scattering by larger particles tends to hide the smaller particle populations, can be overcome by applying it after a size fractionation separation step such as asymmetric field flow fractionation (AF4) (Iavicoli et al., 2015). However, the measurement following AF4 particle separation usually needs much longer analysis times, includes particle specific method development and frequently also dilutes the concentration of particles below the limit of detection especially for smaller particles.

While simple UV-spectrophotometric procedures also exist for determination of DoxHCl concentration (Manasa et al., 2013), liquid chromatographic (LC) methods are the golden standards in the analysis of drug concentration in pharmaceutical products and biological media (Maudens et al., 2009). Various detectors (from UV–vis absorbance to mass spectrometry) coupled to the LC system provide a wide range of specific quantification techniques for the different active compounds – after LC separation method development and calibration.

Although Analytical ultracentrifugation (AUC) is a method mostly applied in protein size measurements and kinetic studies (Brown and Schuck, 2006, Dam et al., 2005, Schuck, 2000) it has also great potential in measuring particle size up to the micrometer range – depending on the density of nanoparticles (Carney et al., 2011, Wohlleben, 2012). AUC is a first-principles based technique, requiring no calibration by means of a particle size standard and determining particle size from the sedimentation speed of the components of a suspension (Mittal et al., 2010, Planken and Colfen, 2010). Modern AUC instruments are capable to monitor sedimentation of polymeric nanoparticles and liposomes in a water based suspension using both absorbance and/or refractive index (RI) detector(s). In case of known densities, the measured sedimentation coefficient distributions can be converted to mass based size distributions. However, even direct comparison of sedimentation coefficient distributions can provide information about batch to batch variability or drug loading.

As the molecular mass of a typical small the drug molecule is generally about 5 orders of magnitude lower than the mass of an encapsulating liposome, sedimentation speed of the free drug is negligible compared to the sedimentation speed of the nanoparticles. In case of molecules absorbing in the UV–vis spectral region, this results in a practically stable time-independent and radius-independent free-drug background absorbance at properly chosen AUC rotational speed. The ratio of this “background” signal to the signal corresponding to the sedimenting fraction(s) provides information about the ratio of free absorbing material outside the liposomes.

In this work, we describe the applicability and limitations of AUC as simple analytical method in the characterization of nanomedicine products. We measure free vs. encapsulated drug ratio and particle size distribution by AUC and demonstrate that the generated results match well with reference method results (HPLC analysis and DLS).

## 2 Materials and methods

### 2.1 Materials

Doxorubicin containing Dox-NP™ and control empty liposomes were purchased from Avanti PolarLipids. According to the manufacturer's specification, nominal doxorubicin concentration was about 2 mg/mL with 97.3% encapsulation in the loaded liposomes. Doxorubicin hydrochloride (DoxHCl, European Pharmacopeia reference standard) and all other chemicals were purchased form Sigma Aldrich. Phosphate buffered saline solution (PBS) was filtered through a 0.2 μm syringe filter (Millipore) before use.

### 2.2 UV–vis spectroscopy

UV–vis (UVVis) spectra of the Dox-NP™ suspension and the free drug were recorded in PBS using 0.5 mL quartz cuvettes and a Nicolet Evolution 300 (Thermo) UV–vis spectrophotometer.

### 2.3 Particle size measurements

Batch mode DLS measurements were performed using a Malvern Zetasizer Nano-ZS instrument equipped with a 633 nm HeNe laser. The original liposome suspensions were diluted 100 times in PBS and equilibrated for 5 min before the measurements at 25 °C. Size distribution results were generated by averaging 10 consecutive measurements of 12 times 10 s runs.

Online coupled FFF-UVVis-MALS-DLS measurements were performed using a Postnova AF4 asymmetric field flow fractionation system connected to a Malvern Zetasizer Nano-ZS instrument and a Wyatt Dawn Heleos multi angle light scattering (MALS) detector. The diluted liposome suspensions were injected through a 20 μL loop into the FFF channel (350 μm spacer, 10 kDa regenerated cellulose membrane). PBS (pH 7.4) was applied as mobile phase at 0.5 mL/min flow, 1.3 mL/min crossflow and exponentially decreasing crossflow profile and total running times of 60–80 min. Absorbance of the eluted fractions was monitored at 490 nm and 280 nm for the loaded and empty liposomes, respectively. Hydrodynamic and geometric size of the particles was determined at the maximum of the elugram from the on-line DLS and MALS measurements, respectively.

Cryo-TEM images were taken using a Tecnai Osiris (FEI) transmission electron microscope at 200 kV. Before sample preparation Dox-NP™ suspension was 1:1 diluted in MilliQ water, while the suspension of control liposomes was loaded on the grid and analyzed without any dilution. A sample aliquot of 2 μL was spot on Agar C-166-3 lacey carbon grid. The sample was automatically vitrified by using a Vitrobot (FEI). The raw images were analyzed manually counting 300 particles for each sample, and collecting size (Feret diameter) and shape information by Image J. Size distributions were plotted by using Origin v 8.0. Average size and standard deviation were calculated by fitting the two histograms with a normal distribution curves.

### 2.4 HPLC analysis

Analysis of doxorubicin concentration was conducted using a Waters liquid chromatographic system composed of a 1525 binary pump and a 2487 dual lambda absorption detector set at 234 nm wavelength. Chromatographic separation was achieved using a Phenomenex Kinetex 2.6 μ XB-C18 100A (75 × 4.6 mm) column. The mobile phase was composed of (A) 0.1% trifluoroacetic acid (TFA) in ultrapure water and (B) 0.1% TFA in acetonitrile. The gradient method was (v/v): 15% B (3 min), 15–100% B (10 min), 100% B (3 min), 100-15% B (4 min), 15% B (10 min), with total runtime of 30 min. Calibration was set up in the 0–40 μg/mL range. Injection volumes of 40 μL were used for both sample and calibration solutions.

Analysis of the Dox-NP™ samples was performed after release of the encapsulated drug by destroying the liposomes with lyophylization and subsequent solubilization with a suitable solvent (75% H2O, 25% ACN). According to our former experience, this solvent mixture ensures complete solubilization of the active ingredient at our working conditions, prevents the formation of micelles and is compatible with subsequent separation by liquid chromatography.

Filtration of the test sample was performed after 1:1 dilution in PBS using 100 kDa cut-off (MWCO) regenerated cellulose Amicon Ultracentrifugal Filters (Millipore) at 5000 rpm, filtering 200 μL suspensions. The pore size of the filtration device (100 kDa corresponding to about 8 nm diameter) used in the HLPC based experiment was one order of magnitude lower than the hydrodynamic diameter of the liposomes. In order to verify that the applied centrifugation force does not destroy the vesicles, a higher speed control sample was centrifuged at 10,000 rpm, followed by comparison of filtrate concentrations and check of particle integrity in the remaining small volume suspension by DLS measurements. Filtration device controls were prepared dissolving known concentrations of free doxorubicin HCl in PBS. Spiked sample was also prepared by addition of free drug at known concentration to the 1:1 diluted sample (in PBS). Total DoxHCl concentration before filtration was determined by HPLC analysis of 20 μL aliquots dissolved in 1 mL solvent (75% H2O, 25% ACN) after freeze drying (in triplicate). DoxHCl concentration in filtrates was determined by diluting 100 μL filtrate to 1 mL in the solvent (75% H2O, 25% ACN). DoxHCl concentration in spiked samples was determined by diluting 20 μL filtrate to 1 mL in the solvent. Integrity of the concentrated samples and vesicle leakage through the filter was evaluated by batch mode DLS measurements.

### 2.5 AUC experiments

A Beckman Coulter ProteomeLab™ XL-I analytical ultracentrifuge was used for the AUC analysis of the samples. For determination of the free versus encapsulated drug ratio, 10 μL aliquot of the original liposome suspension was diluted to 500 μL in PBS. The spiked Dox-NP™ suspension was prepared by diluting 6 μL of Doxil suspension in 382 μL PBS and adding 2 μL of 2 mg/mL DoxHCl. The sample with added empty vesicles contained Dox-NP™ and control vesicle suspensions in equal volumes at 75 x dilution in PBS. The volume of suspension loaded in the sample cell of the centerpiece was 390 μL, while the reference cell contained 400 μL PBS. The sample holders were placed in an 8-hole rotor and measurements were run at a constant rotation speed of 10,000 rpm at 20 °C. Interference and absorbance (490 nm) signals were collected in parallel in the same run. The starting total and final residual baseline absorption signals were calculated from the first and last measurements, averaging 84 absorption data points in the 6.2–6.5 cm radial position range.

Density of the loaded and empty vesicles was estimated by AUC measurements performed at 490 and 280 nm, respectively. Samples were diluted in sucrose solutions with various densities in the 1.02–1.07 g/mL range. Reference cell of the AUC centerpiece was loaded with sucrose solution of the same density. Measurements were run at 40,000 rpm rotational speed.

The ls-g*(s) model of the Sedfit software (Schuck et al., 2002) was applied to fit experimental data (using linear grid in the 1–500 S range) to calculate sedimentation coefficient distributions. Sedimentation coefficient distributions were transformed to mass based size distributions using the “transform s distribution to r distribution” option of Sedfit.

## 3 Results and discussion

### 3.1 Particle size distribution of Dox-NP™

The intensity based size distribution of the loaded liposomes (Figure SM1, Supplementary material) at 100× dilution in PBS as obtained by DLS shows a well-defined peak with a mean of 80 nm, Z-average value of 76 nm and a polydispersity index (PdI) of 0.04. The control vesicles have an intensity mean of 85 nm with Z average of 81 nm and PdI of 0.018. Online coupled FFF-UVVis-MALS-DLS analysis of the samples confirms the batch mode DLS hydrodynamic diameter results and the good monodispersity of the samples (Supplementary material, Figure SM2). The detected size values are in good agreement with the specifications reported by the manufacturer (75 and 82 nm for the loaded and empty vesicles, respectively). Cryo-TEM images Raw and the histogram of size distribution (average radius) obtained for both samples are reported in Figure SM3. The loaded and empty liposomes possess an average diameter of about 57 nm and 63 nm, respectively. Elongated crystals of doxorubicin are present in the interior of the loaded vesicles, which – similarly to the control particles – maintain a spherical shape.

### 3.2 Light absorption behavior of the free and encapsulated doxorubicin

**Fig. 1:**
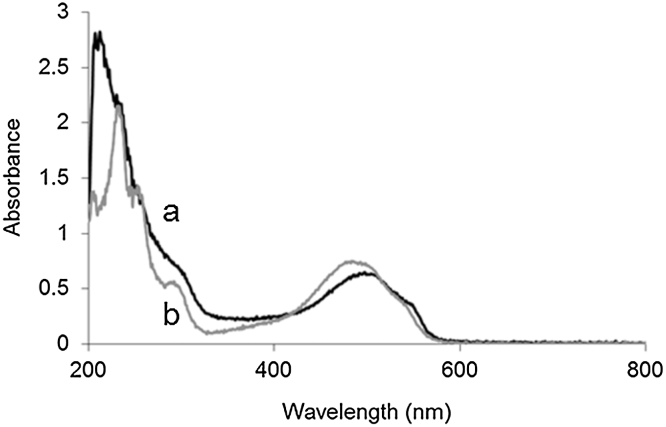
UV–visible spectrum of a) Dox-NP™ liposomes in PBS, b) DoxHCl in PBS at the same (40 μg/mL) doxorubicin concentration.

The absorption maximum of doxorubicin in Dox-NP™ is around 490 nm (Fig. 1a). The spectrum is slightly shifted to higher wavelengths compared to the spectrum of free doxorubicin (Fig. 1a) and the absorbance at 490 nm is lower as well. This behavior is associated to the altered light absorption behavior of aggregated doxorubicin molecules (Changenet-Barret et al., 2013).The comparison between encapsulated and free DoxHCl (at the same 40 μg/mL concentration) shows that the free drug has higher molar attenuation coefficient at 490 nm than the encapsulated (crystallized) form. Because of this, uncorrected absorption based measurements (including AUC) will underestimate the amount of encapsulated drug, resulting in overestimation of the free molecules.

### 3.3 Free versus encapsulated drug ratio measurements by ultrafiltration and HPLC analysis

**Fig. 2:**
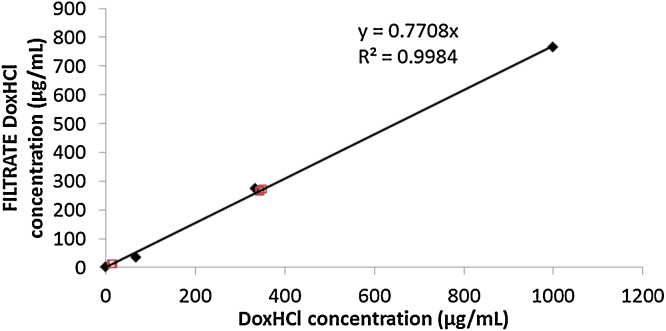
Filtrate DoxHCl concentrations after filtration of solutions with known DoxHCl concentration (black diamonds). The concentration range is selected according to the 1:1 dilution performed before filtration. Open squares represent the sample and spiked sample measurements.

The applied sample preparation (lyophylization and dissolution) and HPLC method (Supplementary material Figure SM4) allow precise quantification of the drug content both in the original sample and the filtrate using appropriate calibration. However, the adsorption of the doxorubicin on the filter device needs to be also quantified in order to determine the loss of the active component during filtration. Therefore, solutions of free drug in PBS were filtered using the same conditions and same type of filter device as for the test sample. The filtrate concentration was found to be linearly dependent from the concentration of the original solution in the investigated (relevant) range with about 77% recovery of DoxHCl concentration in the filtrate and no interfering signal in the blank medium sample (Fig. 2). The higher speed control did not show increased DoxHCl concentration in the filtrate and DLS measurements of the remaining suspension suggested similar particle size distribution as in the original sample confirming particle integrity during the filtration.

Calculating with this concentration-dependent DoxHCl loss, the free drug concentration of the original sample can be determined as follows:

Free drug concentration = 2 (Free drug concentration in Filtrate)/0.7708

where 0.7708 is the slope of the curve in Fig. 2, describing the concentration dependent DoxHCl recovery during the filtration.

Free drug content [%] = (Free drug concentration)/(Total drug concentration)*100

and

Encapsulated drug content [%] = 100 [%]- Free drug content [%]

According to the HPLC measurements and the calculations described above, free drug content (±standard deviation) in the original Dox-NP™ l suspension resulted as being 1.52 (±0.20) % with encapsulated drug ratio of 98.48 (±0.20) %. Standard deviations were calculated considering the error propagation, but not calculating with the uncertainties of the calibrations curves.

In case of the spiked sample, the original free drug and encapsulated drug content was found to be 1.54 (±0.44) % and 98.45 (±0.44) %, showing very good agreement with the values calculated for the non-modified test sample. The encapsulation specified by the manufacturer (97.3%) is very close to the measured values. High speed controls and DLS measurements of the concentrated suspensions indicated that the filtration itself is not destructive for the liposomes and does not trigger detectable release of the encapsulated active compound (results not shown). DLS analysis of the filtrates at the same attenuator selection was not possible because of the lack (or very low concentration) of particles, confirming the right choice of the filter pore size.

### 3.4 Free versus encapsulated drug ratio estimation by analytical ultracentrifugation

**Fig. 3:**
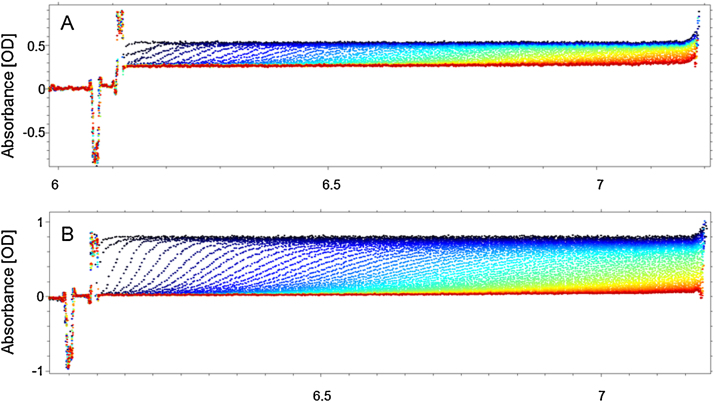
AUC sedimentation profile of A) Dox-NP™ suspension diluted in PBS, spiked with free drug B) Dox-NP™ suspension diluted 50× in PBS. Y-axis is optical absorption at 490 nm while the X axis represents radial positions (distance in cm from center of rotation) in the sample cell. The changing colour of the curves from dark blue to red corresponds to increasing centrifugation time. (For interpretation of the references to colour in this figure legend, the reader is referred to the web version of this article.)

The analysis of AUC absorbance raw data of Dox-NP™ suspensions without the removal of time and radius independent signal (“noise”) provides direct information about the concentration ratio of sedimenting and non-sedimenting (or very slowly sedimenting) species in the samples. The difference between the behavior of the signal originating from the dissolved free drug and from the liposomes can be clearly observed in case of a spiked sample containing free DoxHCl absorbing similar amount of light as the encapsulated drug (Fig. 3A). The sedimentation profile of this sample shows that after the liposomes passed the full length of the cell, a background that is about half of the total signal remains because of the dissolved DoxHCl. On the contrary, the AUC profile of the original Dox-NP™ suspension (diluted without spiking) shows that most of the original total absorption, 0.798 (±0.009) corresponds to the sedimenting particles and only very small signal, 0.020 (±0.005) remains after the vesicles passed (red curve). This signal belongs to the dissolved free DoxHCl that has extremely low sedimentation velocity at the applied rotor speed. As the attenuation coefficient of the doxorubicin inside the vesicles is lower than the attenuation coefficient of free doxorubicin (as shown above by the UV–vis measurement), one simple AUC experiment (at 490 nm) can estimate only the possible maximum ratio of the free drug and the minimal encapsulation in the suspension as follows:

Max free drug content [%] = (Remaining baseline absorption signal)/(Original maximum absorption signal)*100

Min encapsulated drug content [%] = 100 [%]- Max free drug content [%]

Based on these calculations, the maximum free drug content in the Dox-NP™ sample is 2.5 (±0.2) % and the sample contains at least 97.5 (±0.2) % encapsulated drug.

These values are in reasonable agreement with the HPLC results and the manufacturer's specification, and as expected, the AUC measurement at the absorption maximum of doxorubicin slightly underestimates the amount of encapsulated drug compared to the HPLC measurement.

### 3.5 Sedimentation coefficient distribution of liposomes by analytical ultracentrifugation

**Fig. 4:**
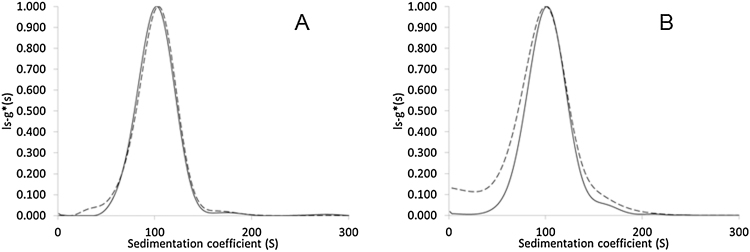
Normalised ls-g*(s) sedimentation coefficient distributions based on absorption (solid line) and interference (dashed line) measurements. A) Loaded Dox-NP™ vesicles, B) Mixture of loaded and empty vesicles.

After fitting the absorption or interference data using the ls-g*(s) model of the Sedfit software, sedimentation coefficient distribution of the particles can be calculated. These distributions might be used for quality control purposes even without transforming them to size distribution curves. Fig. 4A illustrates the excellent match of the normalized differential sedimentation coefficient distributions calculated from the absorbance and interference signal for the Dox-NP™ suspension compared to the mismatch in a sample that contained added control (empty) vesicles.

As the empty vesicles are expected to have slightly lower density compared to the loaded ones, the sedimentation speed of these particles is lower than the velocity of particles containing the encapsulated drug (Fig. 4B). The absorption detector at 490 nm – specific for DoxHCl – is sensitive only for loaded vesicles, while the interference optics detects both loaded and empty particles. In our case, the shoulder of the interference based sedimentation coefficient distribution curve at the lower values (Fig. 4B) could be indicative the presence of unloaded vesicles or lipid fragments in the suspension.

### 3.6 Particle density by analytical ultracentrifugation

**Fig. 5:**
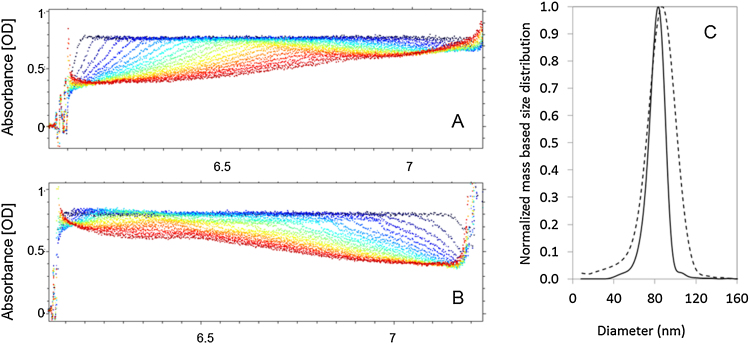
AUC sedimentation profile of Dox-NP™ suspension in A) sucrose solution with density of 1.05 g/mL, B) sucrose solution with density of 1.06 g/mL. Y-axis is optical absorption at 490 nm while the X axis represents radial positions (distance in cm from center of rotation) in the sample cell. The changing colour of the curves from dark blue to red corresponds to increasing centrifugation time. C) Mass based size distribution of Dox-NP™ (solid line) and empty vesicles (dashed line) calculated from AUC measurement using interference optics. (For interpretation of the references to colour in this figure legend, the reader is referred to the web version of this article.)

Sedimentation coefficient distributions generated using the interference optics can be directly transformed to mass based size distributions because of the linear correlation between concentration and refractive index change (Cole et al., 2008). However, density of the particles as input value (besides the density and viscosity of the fluid) is needed for the calculations. AUC sedimentation profiles of the loaded liposomes (Fig. 5) show, that about 50% of the particles are sedimenting at a fluid density of 1.05 g/mL (Fig. 5A) and that about 50% of the particles is moving in the meniscus direction (Fig. 5B) at a fluid density of 1.06 g/mL. (Shown for more density values in Supplementary material, Figure SM6.)

Based on this finding, density of the loaded vesicles was estimated to be around 1.055 g/mL, while empty vesicles were found to have a slightly lower density of about 1.045 g/mL (Supplementary material, Figure SM5). This is in agreement with our former assumption about the difference in particle density causing the shift between the absorbance and interference based sedimentation coefficient distributions in mixed liposome samples (Fig. 4).

### 3.7 Particle size distribution of liposomes by analytical ultracentrifugation

Interference based detection allows the measurement of sedimentation coefficient distributions even if the liposomes do not have a strong absorption in the UV–vis region, like Dox-NP™. As the refractive index change varies linearly with the concentration change, sedimentation coefficient distributions can be directly converted to mass based size distributions (Fig. 5C). The calculated size distribution mode of the Dox-NP™ particles shows good agreement with the FFF-DLS diameter results indicating a diameter of about 84 nm.

## 4 Conclusion

Analytical ultracentrifugation, originally born for the determination of protein mass from sedimentation speed, has expanded to a much wider field since its discovery. AUC can be used to determine also the sedimentation coefficient distribution and size distribution of nanoparticles, including liposomes. The interference optics allows also the detection of particles with no specific absorption band in the UV–vis region like non-loaded vesicles or vesicles loaded with non UV–vis active molecules. AUC gives very similar size results to FFF-DLS without the need for separation method development (Ross et al., 2015). AUC characterization of nanomedicine products does not require complicated sample preparation either, only possible dilution of the original suspension. Unfortunately, dilution might affect drug release, but other analytical methods usually also require prior sample dilution in a buffer. In case of HPLC analysis after filtration, appropriate dilution was needed to allow proper estimation of the drug adsorbed on the filtration device. Other separation strategies like gel chromatography also result in diluted suspensions.

Besides measuring particle size distributions, AUC is able to provide information on free vs. encapsulated drug ratio if the entrapped molecule has a specific absorption band in the UV–vis region. As demonstrated here for doxorubicin, the raw sedimentation profile data can be directly analyzed to estimate the ratio between the absorbing species in the fast sedimenting liposomes and the slowly sedimenting free drug. The systematic error generated because of the attenuation coefficient difference between the dissolved and entrapped (in this case also crystallized) molecules might result in false positives in a quality control procedure overestimating the amount of free drug, but still keeps the passing samples on the “safe side”. AUC analysis at a wavelength value where the attenuation coefficients are matching might provide even more accurate relative quantification in one single run, without the need of calibration and prior separation step (Supplementary material, Figure SM7). As an additional feature, by the combination of the two (refractive index and absorbance) detection systems, AUC is able to distinguish empty vesicles from loaded ones, and able to detect the presence of non-loaded vesicle population in a mixture of liposomes. AUC instruments with fluorescence optics are also available, further broadening the choice of detection methods in case of sensitivity issues or samples with specific fluorescent signal.

The unique capability of AUC in providing data for size and composition analysis in a single measurement without complex sample preparation makes this characterization method promising tool on both the product development and quality control fields.
