# Supplementary material for: Analytical ultracentrifugation for analysis of doxorubicin loaded liposomes
Source: Int J Pharm. 2017 May 15;523(1):320–6. doi: 10.1016/j.ijpharm.2017.03.046 (PMC5405781; doi:10.1016/j.ijpharm.2017.03.046)
Supplement: Supplementary file 1 [file mmc1.docx]

**Supplementary material**

DLS

Figure SM1: Batch DLS, intensity based size distribution of the Dox-NP^TM^ liposomes (red) and control liposomes (green) at 100 x dilution in PBS. Averages of 10 measurements.

FFF-UVVis-MALS-DLS


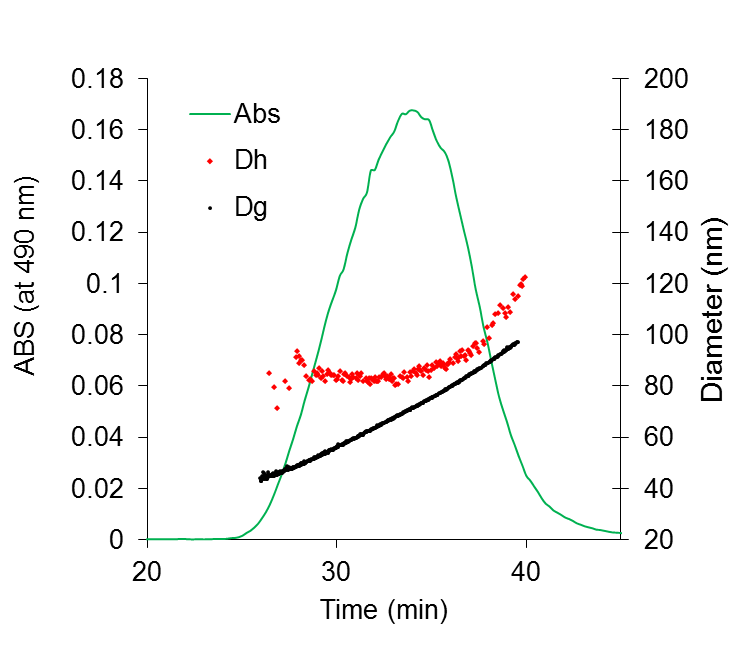

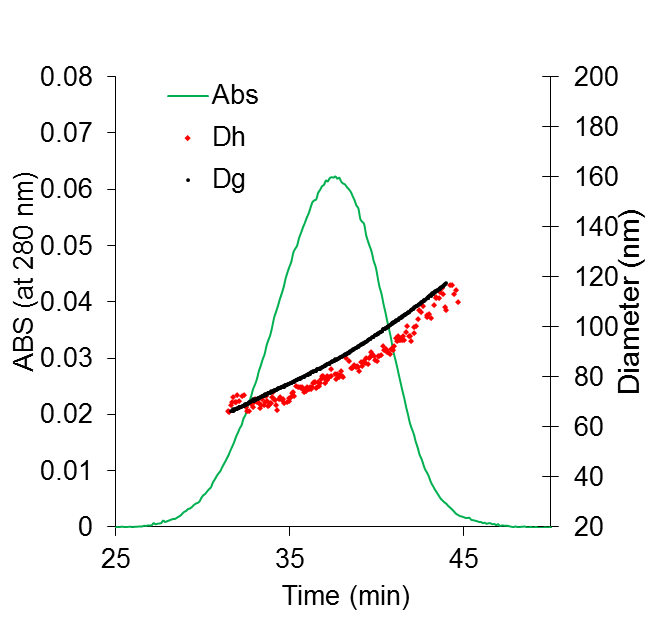


A B

Figure SM2: FFF elugram (absorbance) peak (green), hydrodynamic diameter by DLS (red) and geometrical diameter by MALS (black) of the loaded (A) and empty (B) liposomes

Cryo-TEM


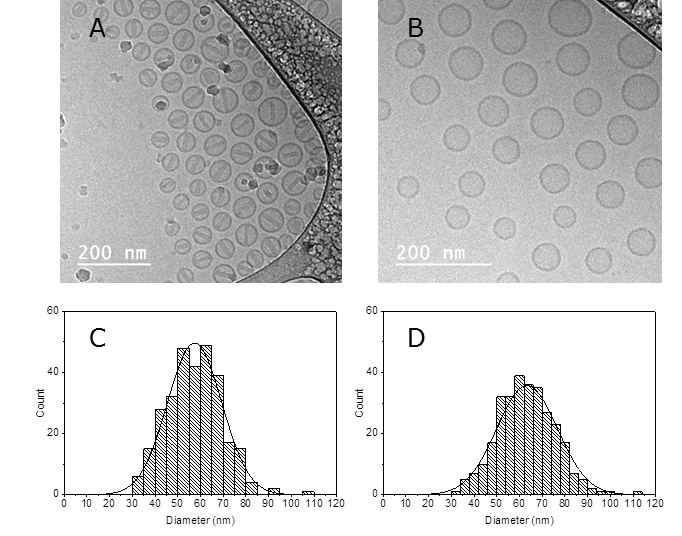


Figure SM3: Cryo-TEM images and corresponding size distributions of (A,C) loaded and (B,D) empty liposomes.

HPLC

**
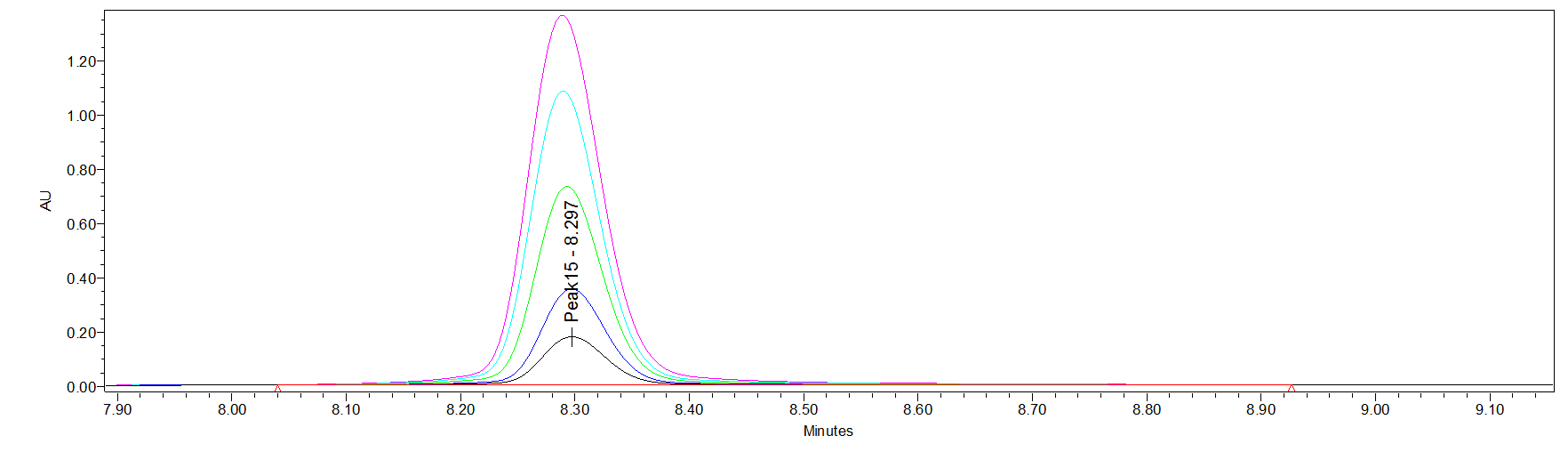

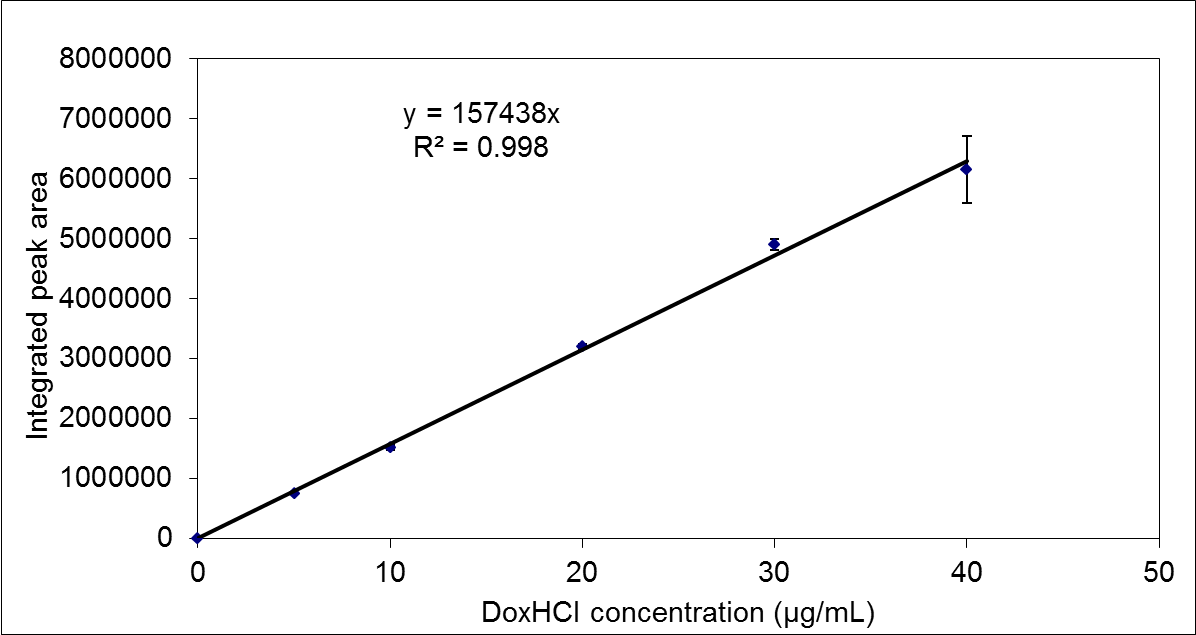
**

Figure SM4: HPLC DoxHCl calibration curve and standard chromatogram peaks (insert)

AUC
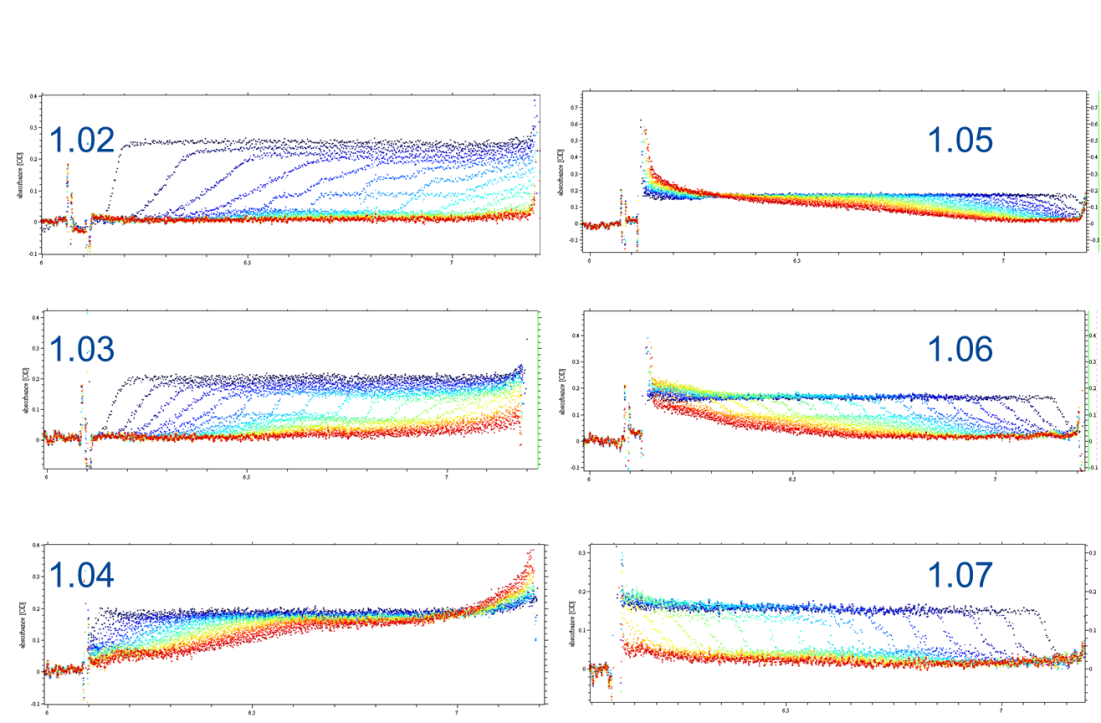


Figure SM5: AUC sedimentation profile of Doxil control (empty liposome) suspension in various density sucrose solutions. Y-axis is optical absorption at 280 nm while the X axis represents radial positions (distance in cm from center of rotation) in the sample cell. The changing colour of the curves from dark blue to red corresponds to increasing centrifugation time.


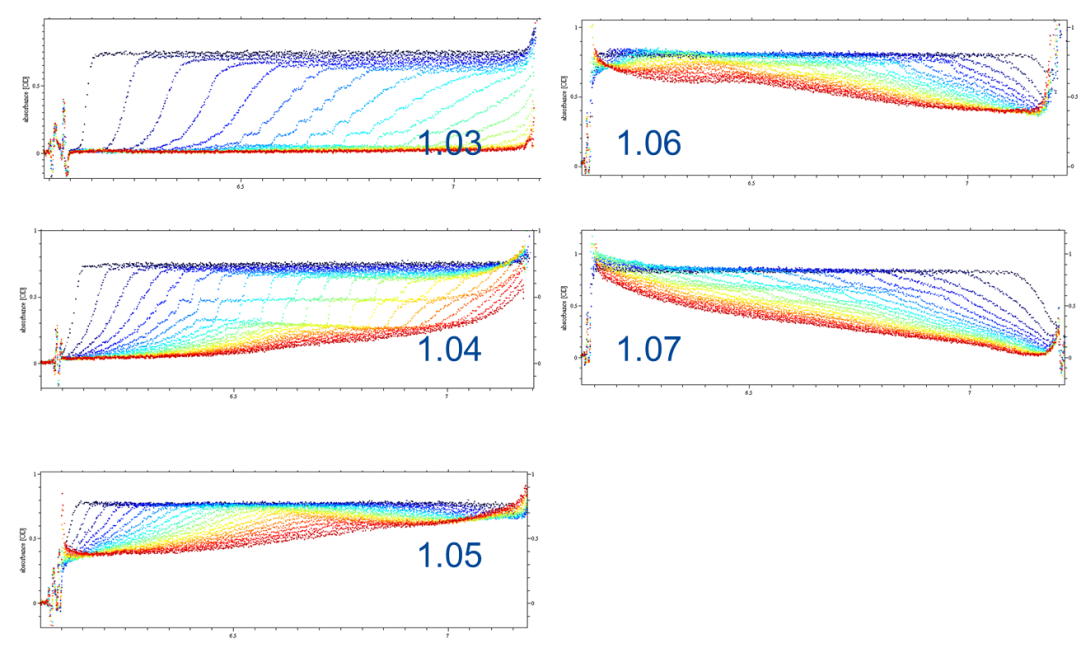


Figure SM6: AUC sedimentation profile of Doxil (loaded liposome) suspension in various density sucrose solutions. Y-axis is optical absorption at 490 nm while the X axis represents radial positions (distance in cm from center of rotation) in the sample cell. The changing colour of the curves from dark blue to red corresponds to increasing centrifugation time.


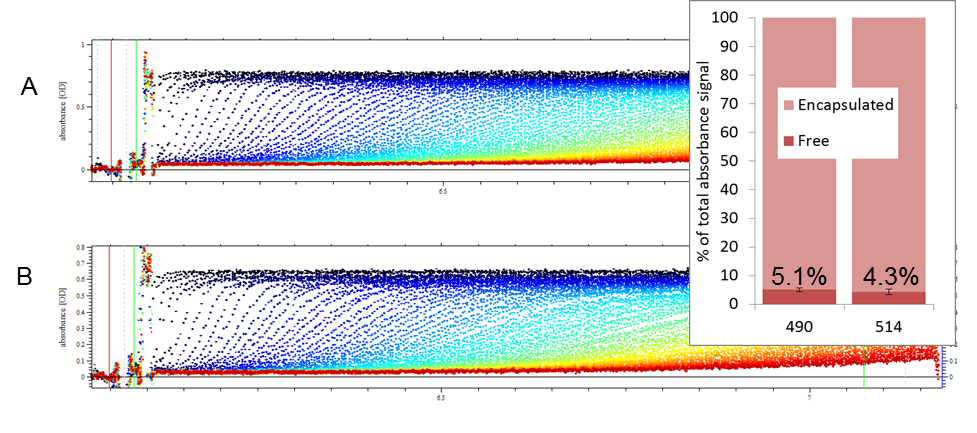


Figure SM7: Sedimentation profiles of a Doxil suspension A) at 490 nm, B) at 514 nm. Insert: Free drug content based on the measurements at 490 and 514 nm.
